# Comparative Analysis of Structural Efficiency of Steel Bar Hyperbolic Paraboloid Modules

**DOI:** 10.3390/ma18174127

**Published:** 2025-09-02

**Authors:** Jolanta Dzwierzynska, Patrycja Lechwar

**Affiliations:** Faculty of Civil and Environmental Engineering and Architecture, Rzeszow University of Technology, Al. Powstancow Warszawy 12, 35-959 Rzeszow, Poland; p.lechwar@prz.edu.pl

**Keywords:** steel structures, bar structures, finite element method, parametric design, structural analysis and optimization, modular structures, generative design, manufacturing

## Abstract

Curved roofs constructed using hyperbolic paraboloid (HP) modules are gaining popularity in structural engineering due to their unique aesthetic and structural advantages. Consequently, these studies have investigated steel bar modules based on HP geometry, focusing on how variations in geometric configuration and bar topology affect internal force distribution and overall structural performance. Each module was designed on a 4 × 4 m square plan, incorporating external bars that formed the spatial frame and internal grid bars that filled the frame’s interior. Parametric modeling was conducted using Dynamo, while structural analysis and design were performed in Autodesk Robot Structural Analysis Professional (ARSAP). Key variables included the vertical displacement of frame corners (0–1.0 m at 0.25 m intervals), the orientation and spacing of internal bar divisions, and the overall mesh topology. A total of 126 structural models were analyzed, representing four distinct bar topology variants, including both planar and non-planar mesh configurations. The results demonstrate that structural efficiency is significantly influenced by the geometry and topology of the internal bar system, with notable differences observed across the various structural types. Computational analysis revealed that asymmetric configurations of non-planar quadrilateral subdivisions yielded the highest efficiency, while symmetric arrangements proved optimal for planar panel applications. These findings, along with observed design trends, offer valuable guidance for the development and optimization of steel bar structures based on HP geometry, applicable to both single-module and multi-module configurations.

## 1. Introduction

The hyperbolic paraboloid (HP) is among the most distinctive forms of curved spatial surfaces [1,2,3,4]. Its geometry presents unique opportunities for the development of structural systems that harmonize architectural aesthetics, functional performance, and material efficiency. Owing to its characteristic double curvature, the HP exhibits exceptional capabilities in load distribution, making it particularly well-suited for long-span applications where lightweight yet high-strength structural solutions are essential [5].

The architectural and engineering exploration of HP structures began in the mid-20th century and was led by pioneers such as Félix Candela, Eduardo Torroja, and Heinz Isler, who experimented with thin-shell reinforced concrete designs [6]. Their work gave rise to structures of exceptional elegance and minimalist design, many of which continue to be revered as enduring exemplars of structural artistry. Félix Candela, in particular, became renowned for his mastery in composing complex and expressive forms from simple reinforced concrete HP segments [7,8,9,10].

The modern approach to curved structures increasingly favors steel structures [11,12], which offer significantly greater possibilities for prefabrication, lightness, and precision than structures made from other materials [13]. As the HP surface is a ruled surface, any curved steel structure in the form of the HP can be made of straight members, in particular of a spatial frame filled with bars [14]. This configuration provides high overall stiffness and structural stability while optimizing material usage [15,16,17,18,19]. Steel bar structures forming HP grids can be manufactured with high accuracy in factory conditions and then assembled on-site in a very short time [14]. This allows for significant labor cost savings and reduces the overall construction schedule.

Investigations into hyperbolic paraboloid-based structures date back to the mid-20th century, when their unique geometric and structural properties attracted significant scholarly attention [20,21]. Previous research on these structural systems has predominantly concentrated on evaluating strength, stability, and material efficiency [22,23], with particular emphasis on performance under dynamic and thermal loading conditions [24,25,26,27,28,29,30]. In contemporary studies, advanced computational methods [31,32]—especially finite element analysis (FEA)—have facilitated highly accurate modeling of structural behavior, enabling researchers to account for complex surface geometries and interaction among individual components. Increasingly, the shaping of geometry and topology, as well as the analysis of structures, is carried out using genetic algorithms and optimization [33].

Given their exceptional adaptability to diverse architectural forms and engineering constraints, HP structural systems offer significant design flexibility. This inherent versatility facilitates the adoption of innovative construction approaches, including modular design strategies [34,35,36,37]. However, the adoption of modular systems enables the development of structures at various scales while facilitating efficient assembly and disassembly—an advantage particularly valuable in fast-track construction projects.

A noteworthy aspect of designing steel bar structures based on the HP is the possibility of discretizing the HP surface into flat quadrilateral panels, which means finding a polyhedron with quadrangular faces that could replace the curvilinear shape. A similar approach was used in the case of the dome shape in [38,39]. Thanks to this opportunity, a complex HP double-curved surface can be approximated by simple planar elements, which significantly simplifies both structural analysis, fabrication, and enhances assembly efficiency. Furthermore, it supports the integration of flat panel systems such as photovoltaic glazing—an increasingly important aspect for sustainable engineering design [37].

Given the aforementioned advantages of HP-shaped steel bar structures and their considerable potential for modular applications [40], this study aims to conduct a comparative evaluation of representative HP steel bar modules. Specifically, the research investigates whether it is feasible to assess the structural efficiency of a module during the early stages of the design process. If so, the objective is to compare the performance of various steel bar modules based on their geometric shape and the topology of their bar arrangements. This study further explores how modifications in topology influence the structural behavior and internal force distribution within the module bars. An additional focus is placed on evaluating how the insights gained from this analysis can inform the design of more complex structural systems.

## 2. Shaping the Geometry and Topology of the Modules

**Figure 1:**
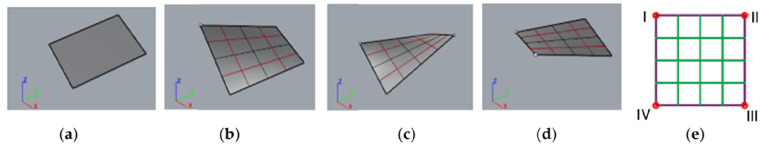
Creation of a HP module: (**a**) output square; (**b**) rising one vertex; (**c**) rising two opposite vertices; (**d**) rising two adjacent vertices; (**e**) horizontal projection of the resulting module.

Geometrically, an HP shape can be obtained from a flat square by appropriately modifying its surface, raising or lowering its vertex/vertices, to obtain a non-flat/spatial surface (Figure 1a–d). This condition will be satisfied provided that no pair of surface sides are parallel. Given that the HP is a ruled surface, it is possible to construct a grid of linear elements by appropriately subdividing its boundary edges. The obtained grid of bars together with the spatial frame limiting it form an HP module. The horizontal view of an example module is shown in Figure 1e.

This study was conducted using grid modules organized on a 4.0 × 4.0 m layout. In each configuration, the primary load-bearing component comprised a quadrilateral frame, within which a secondary internal grid of bars was embedded. This internal grid functioned to collect loads from the cladding and efficiently distribute them to the main structural frame. The variable geometries of the structural frames were generated parametrically through the use of Dynamo, integrated with Autodesk Robot Structural Analysis Professional 2025 (ARSAP) software [41], enabling precise control over design variations and structural performance analysis.

Spatial frame configurations were derived by selectively manipulating the Z-coordinates of designated vertices, resulting in the elevation or depression of specific corners of the structure. The variations included the following:

- Elevation of a single corner (IV), leading to asymmetrical configurations, Figure 1e;
- Elevation of two opposite corners (II and IV), producing either symmetrical or asymmetrical configurations, Figure 1e;
- Elevation of two adjacent corners (III and IV), leading to asymmetrical configurations, Figure 1e.

These geometric transformations enabled the exploration of diverse spatial forms and their structural implications.

**Figure 2:**
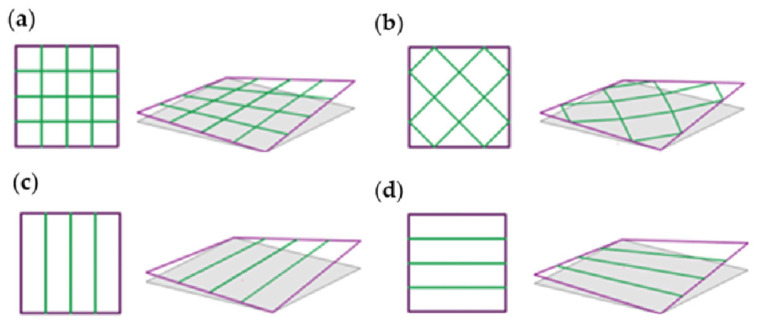
Analyzed topologies of the spatial HP module structures of (**a**) type 1; (**b**) type 2; (**c**) type 3; (**d**) type 4.

The vertical displacement of the vertices ranged from 0.00 to 1.00 m, applied in increments of 0.25 m. The structural variants differed in the configuration of the internal bars within the primary frame. A quadrilateral grid was used, consisting of both flat and non-flat polygons (Figure 2a,b), as well as two types of configurations in which the number of internal bars was reduced to those lying in planes parallel to one of the sides of the frame (Figure 2c,d). They were called structures of type 1, type 2, type 3, and type 4, respectively (Figure 2). The initial structures for each group, in which all vertices are located at the same height equal to 0.00 m, were also included in the analysis.

**Figure 3:**
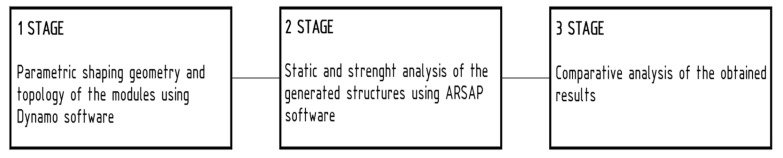
A flow chart of the study.

The study consisted of three stages. The first stage involved shaping the geometry and topology of the structures using Dynamo software. A total of 126 structural models, each with plan dimensions of 4.0 × 4.0 m, were generated and analyzed. These models varied in both the overall geometric configuration of the system and the topology of the internal structural grid. The second stage consisted in static and strength analysis of all generated structures using ARSAP, whereas the third stage involved compiling the results of optimization and structural design, as well as a comparative analysis of the considered models (Figure 3).

**Figure 4:**
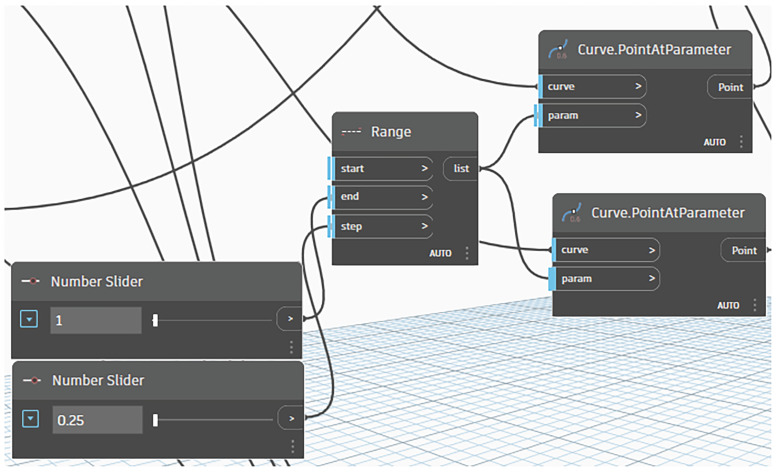
A section of the script defining the internal bar arrangement.

The variable geometries of the structural modules were generated through the integration of Dynamo with ARSAP. However, a custom Dynamo script facilitated the automated manipulation of key design parameters, including the following:

- Support height at one or two corners of the structural frame, defined by the Z-coordinate, with values ranging from 0.00 to 1.00 m in 0.25 m increments;
- Configuration of internal frame bars, encompassing the method and direction of subdivision, as well as the spacing between individual members (Figure 4).

This parametric approach enabled efficient generation and evaluation of multiple structural variants within a controlled design space.

**Figure 5:**
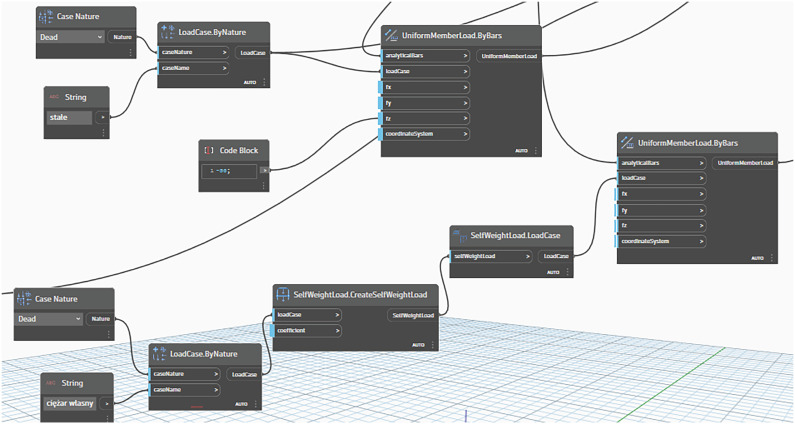
A section of the script specifying the loading conditions of the structure.

To ensure the comparability of the analyzed structures, a set of parameters was defined and held constant across all systems. These included the following:

- Material of structural elements—S235 steel (Figure 5);
- Types of supports and joints;
- Bar loading cases.

The dead load of the steel structure and the cladding load were assumed to be identical across all configurations. The self-weight of each structure was automatically accounted for in the analysis (Figure 5).

The primary objective of the research was to assess the impact of structural geometry and topology on the internal force values and distributions assuming identical loading conditions for all structures.

The entire process of geometry shaping and load application was continuously monitored in ARSAP. As a result of the geometry shaping, structural models were obtained, described in the next subsections.

### 2.1. Type 1 Structure Characteristics

**Figure 6:**
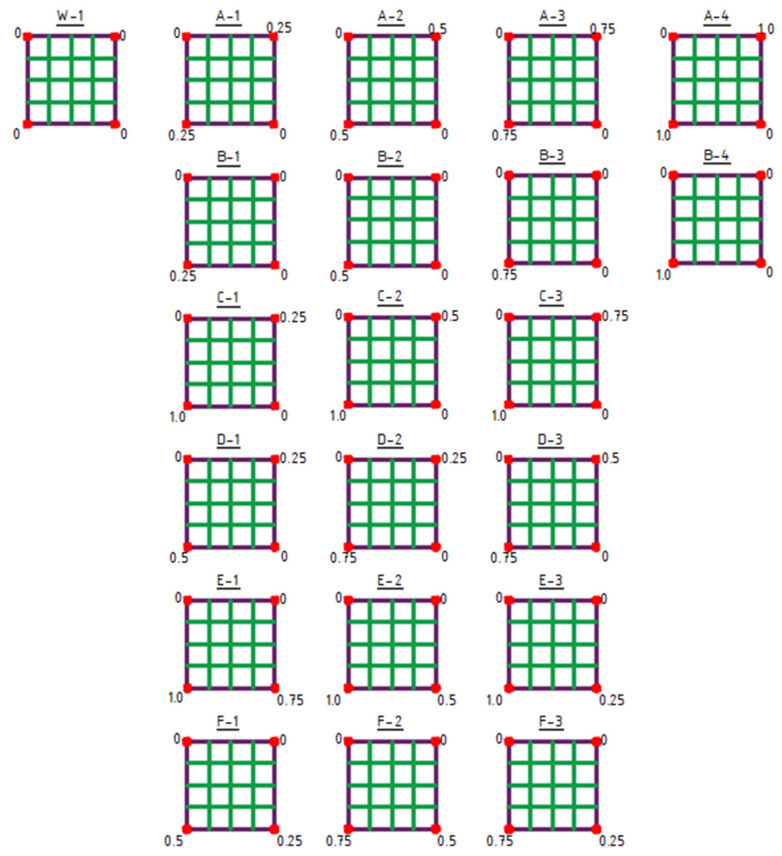
Type 1 structures. The values indicated at the corners represent the Z-coordinate in meters.

The initial structure for shaping type 1 structures was the W-1 structure (Figure 6).

Type 1 structures are characterized by a structural bar arrangement shaped by dividing the sides of the frame into four equal segments (Figure 6).

The first row (structures A-1 to A-4) presents symmetrical structures formed by equally raising two opposite corners of the frame.

The second row (structures B-1 to B-4) displays asymmetrical structures resulting from raising of a single frame corner.

The following two rows illustrate asymmetrical structures generated by the uneven raising of two opposite corners of the frame (structures C-1 to D-3).

Finally, the last two rows show asymmetrical structures formed as a result of the uneven raising of two adjacent corners of the frame (structures E-1 to F-3) (Figure 6).

### 2.2. Type 2 Structure Characteristics

**Figure 7:**
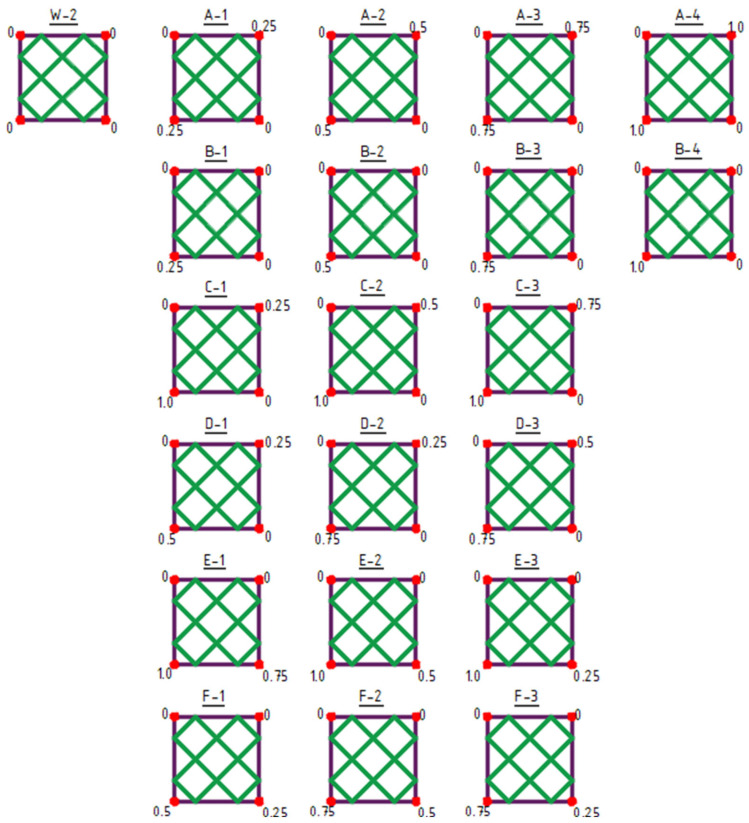
Type 2 structures. The values indicated at the corners represent the Z-coordinate in meters.

The initial structure for shaping type 2 structures was the W-2 structure (Figure 7).

Type 2 structures feature a structural bar arrangement created by subdividing the surface into fields forming flat polygons (Figure 7). The first row (structures A-1 to A-4) presents symmetrical structures formed by equally raising two opposite vertices of the frame.

The second row (structures B-1 to B-4) displays asymmetrical structures resulting from the raising of a single corner of the frame.

The following two rows illustrate asymmetrical structures resulting from the unequal raising of two opposite corners of the frame (structures C-1 to D-3).

The final two rows show asymmetrical structures created by the unequal raising of two adjacent corners of the frame (structures E-1 to F-3) (Figure 7).

### 2.3. Type 3 Structure Characteristics

**Figure 8:**
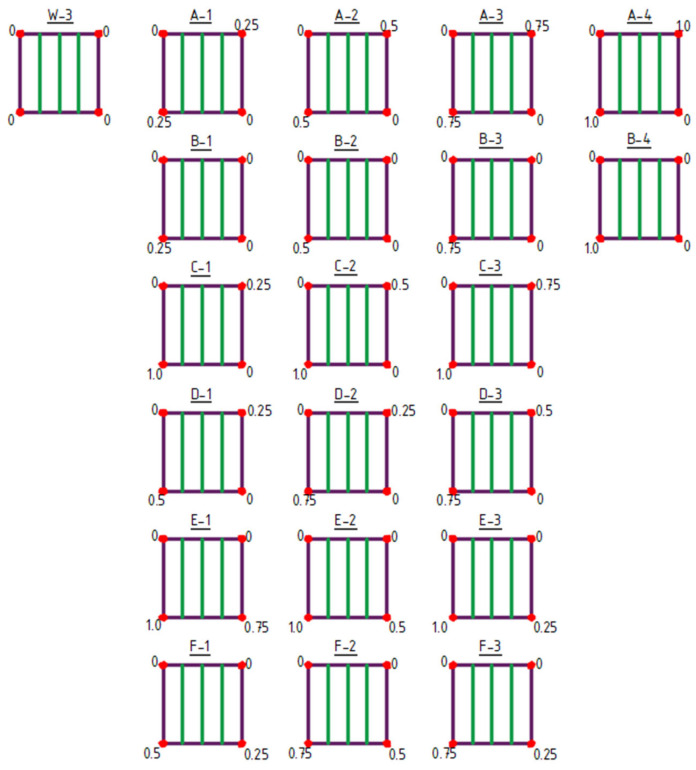
Type 3 structures obtained by raising of vertices. The values indicated at the corners represent the Z-coordinate in meters.

**Figure 9:**
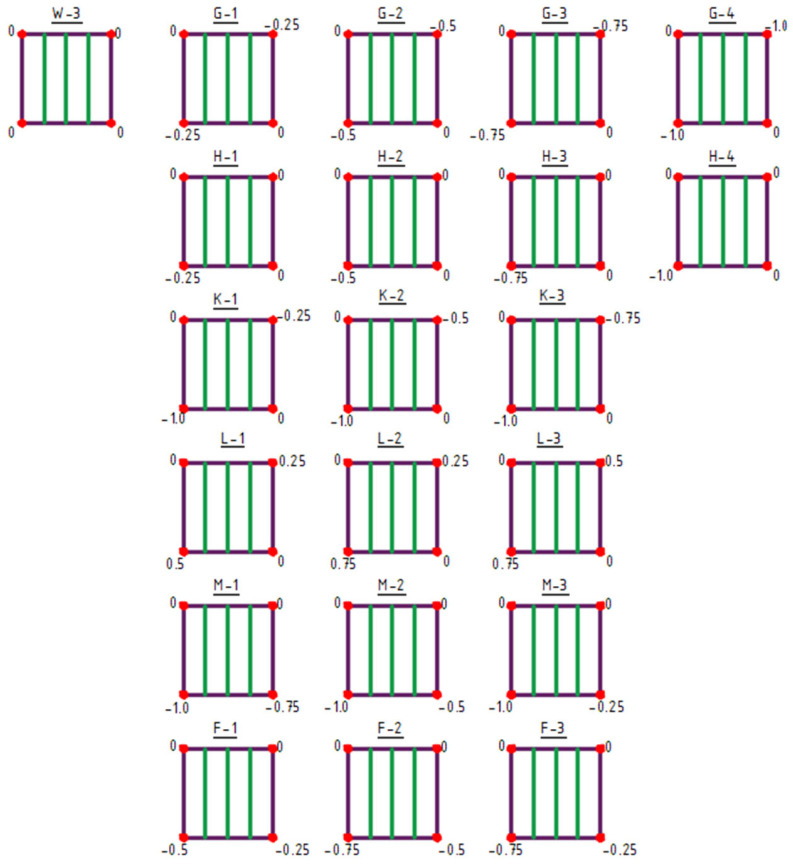
Type 3 structures obtained by lowering of vertices. The values indicated at the corners represent the Z-coordinate in meters.

The initial structure for shaping type 3 structures was the W-3 structure (Figure 8 and Figure 9).

Type 3 structures feature structural bar arrangements in which the number of bars was reduced to a single direction, specifically bars oriented vertically in the plan view (Figure 8 and Figure 9). Within this group, both the raising of vertices (structures A-1 to F-3), Figure 8, and the lowering of vertices (structures G-1 to N-3) were analyzed (Figure 9).

### 2.4. Type 4 Structure Characteristics

**Figure 10:**
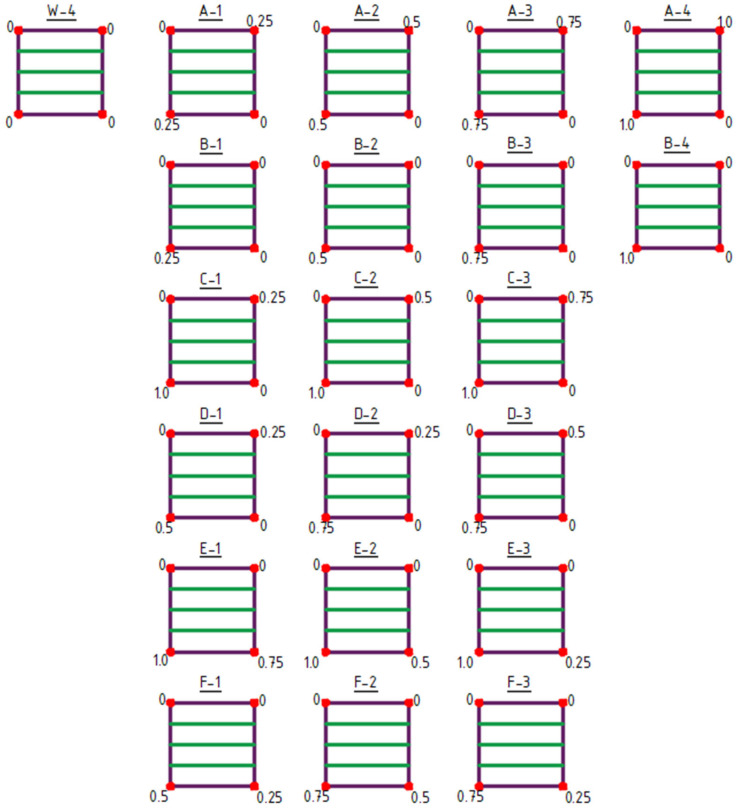
Type 4 structures obtained by raising of vertices. The values indicated at the corners represent the Z-coordinate in meters.

**Figure 11:**
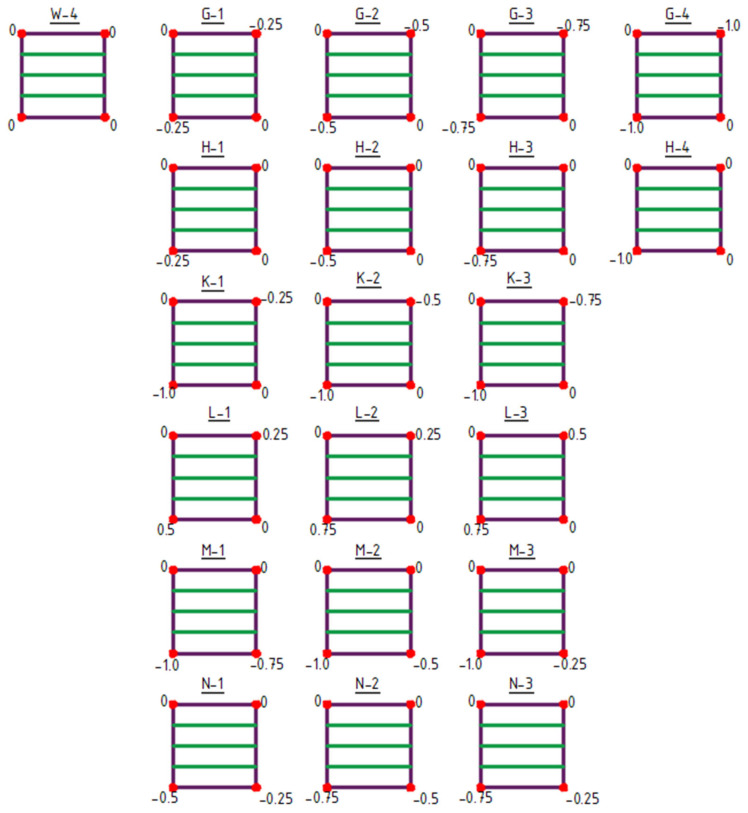
Type 4 structures obtained by lowering of vertices. The values indicated at the corners represent the Z-coordinate in meters.

The initial structure for shaping type 4 structures was the W-4 structure (Figure 10 and Figure 11).

Type 4 structures feature a structural bar arrangement in which the number of bars was reduced to a single direction, with the bars oriented horizontally in the plan view (Figure 10 and Figure 11). Within this group of structures, both the raising of vertices (structures A-1 to F-3) (Figure 10) and the lowering of vertices (structures G-1 to N-3) (Figure 11) were analyzed.

**Figure 12:**
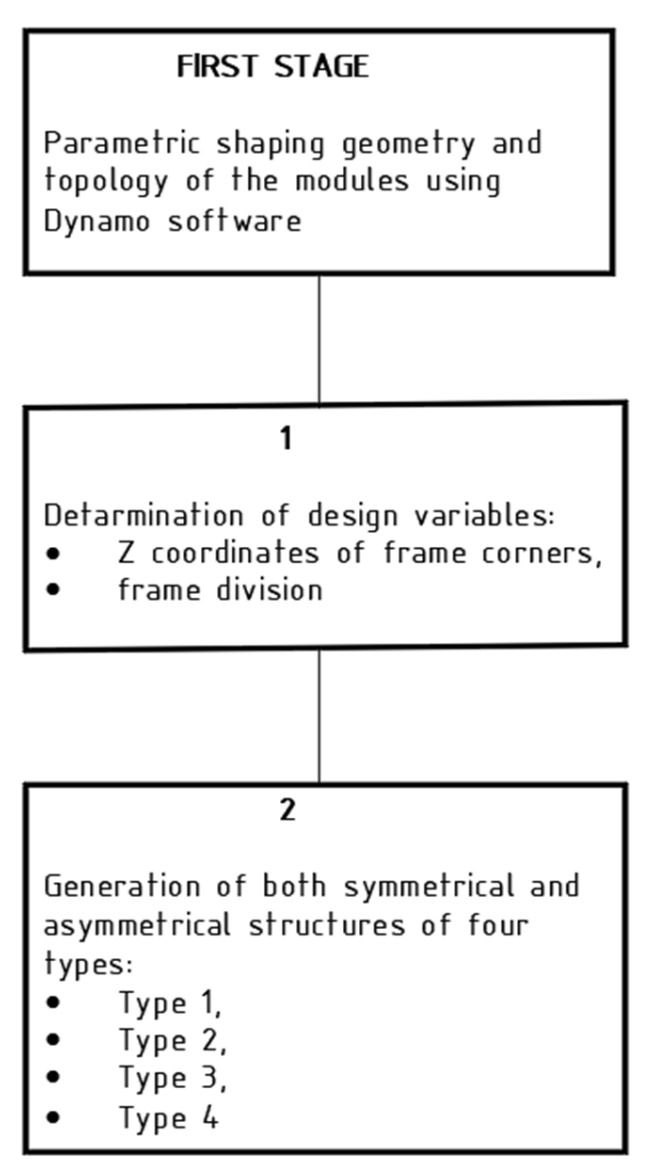
Flow chart of the first stage of the study.

In Section 2, the first stage of research was carried out according to the scheme presented in Figure 12.

A total of 126 geometric models representing four distinct structural types—designated as types 1, 2, 3, and 4—were generated. Under the established initial assumptions, this set of models encompassed the range of possible geometric and topological variations. Identical structures created according to this principle were eliminated. Following the application of supports and loading conditions, these models underwent structural analysis, as detailed in Section 3.

## 3. Structural Analysis and Results

**Figure 13:**
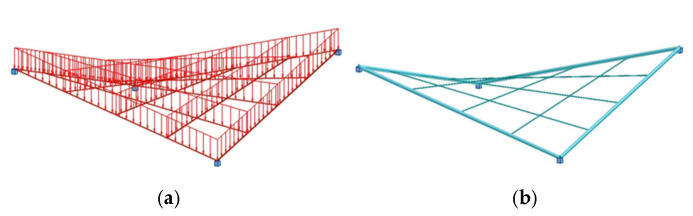
View of the structural model: (**a**) applied loading; (**b**) optimized structure.

As stated earlier, static and strength analysis of the structural model were performed using ARSAP 2025 software (Figure 13). A first-order linear analysis was performed. For the purposes of analysis, a linear bar load was applied, accounting for the cladding weight (assumed as for a metal sheet with a weight of 0.10 kN/m^2^), while the self-weight of the structural bars was automatically included (Figure 13a).

For optimal structural dimensioning, the members were categorized into two distinct groups based on their functional roles within the overall system. One group (G1) consisted of the external bars forming the primary frame load-bearing system. The second group (G2) included internal grid bars created by subdividing the external bars (Figure 13b). The bar types were defined by specifying their buckling factors. All connections between bars were assumed to be rigid. Fixed supports were introduced at the corners. For the applied permanent loads, design load combinations for Ultimate Limit States (ULS) and Serviceability Limit States (SLS) were generated. The next stage involved dimensioning of the members considering ULS and SLS, according to standards [42,43,44]. The selection of the profiles was limited to circular hollow sections, and an optimization option was applied to minimize the structure’s mass. A minimum wall thickness of 4.0 mm for circular profiles was assumed to prevent the ARSAP software from using thin-walled sections.

The static and strength analysis enabled the extraction of results for each structure under identical loading conditions. These results included the following:

- Frame profile utilization (FU) and grid profile utilization (GU);
- The structure’s mass (M);
- Maximum bar deflections (De);
- Maximum node displacements (Di);
- Maximum internal force values (My, Fx, Fz);
- Maximum normal stresses (Fx/A).

The results of the structural and strength analysis, as well as dimensioning, are presented in the following tables:

- Table 1—for structures of type 1;
- Table 2—for structures of type 2;
- Table 3—for structures of type 3;
- Table 4—for structures of type 4.

In the case of type 1 structures, the same cross-sectional profiles were consistently assigned to each bar group across all variants: G1—RO 60.3 × 4.0 mm, G2—RO 25.0 × 4.0 mm. In Polish structural design terminology, the designation RO corresponds to CHS (Circular Hollow Section). The resulting stress levels were similar across the models, and the structures exhibited a significant load-bearing reserve with respect to the ULS (Table 1). However, although the bars were subjected to relatively low internal forces, the chosen cross-sectional profiles were the minimum allowable. Further reductions in size resulted in structural instability.

For type 2 structures as with type 1, consistent cross-sectional profiles were assigned to each bar group across all variants: G1—RO 60.3 × 4.0 mm and G2—RO 25.0 × 4.0 mm (Table 2). Although the internal forces obtained from the static analysis were relatively low, the selected profiles represented the minimum acceptable dimensions.

However, for type 3 structures, identical cross-sections for all bars in all cases were selected: G1 and G2—RO 60.3 × 4.0 mm, Table 3. Despite the low internal forces observed in the bars during static analysis, the adopted cross-sections represented the minimum feasible dimensions from a structural standpoint. Any further reduction in profile sizes resulted in a loss of global or local stability. For type 3 structures, an extended analysis was conducted, which included a larger set of geometric variants. The study included configurations obtained by increasing the Z-coordinate of selected vertices (structures labeled as A-1–F-3), as well as corresponding configurations resulting from a reduction in the Z-coordinate of the same points by an identical magnitude (G-1–N-3). The results of the structural analysis for these types of structures are given in Table 3.

For type 4 structures, as with type 3 ones, identical cross-sections were selected for all bars in every case: G1 and G2—RO 60.3 × 4.0 mm (Table 4). As in the case of other types of structures, the minimum cross-sections for the bars that meet the boundary conditions were selected. Following the same approach used for type 3 structures, the type 4 group was also analyzed using geometries generated by both raising and, alternatively, lowering the vertices by an equivalent magnitude. The results of the structural analysis for these types of structures are given in Table 4.

The results of the structural analysis for the individual models presented in Table 1, Table 2, Table 3 and Table 4 will become the basis for further analysis and evaluation of the structures presented in Section 4.

## 4. Discussion

Based on the obtained results, the considered structures were compared, and their efficiencies were analyzed.

### 4.1. Type 1 Structures

The results presented in Table 1 refer to type 1 structures with an internal bar arrangement forming non-planar meshes.

During the shaping process, the maximum stress utilization was not attained due to relatively low applied loads. Nevertheless, the selected cross-sectional profiles were the minimum allowable. Further reduction in size resulted in structural instability.

Given that all bars in both group G1 and G2 were designed with identical profiles, the total mass of each structure is comparable and does not serve as a decisive criterion for structural efficiency (the difference between models is merely 3 kg). Similarly, maximum deflection, bending moments, and shear forces show no consistent trends and remain at similar levels. The only exception in terms of maximum deflection is the reference structure W-1 (with all corners at the same height), where significantly greater maximum deflection is observed—up to three times higher than in other configurations. However, other internal force values remain at a similar level.

An upward trend can be observed in axial force values and normal stresses. For the reference structure W-1, these values are zero, but as the corners are elevated, axial forces and normal stresses begin to increase. Therefore, these two parameters—axial force and normal stress—can be considered the primary comparison criteria: the greater the height difference between corners, the higher these values. It is also noticeable that symmetric structures tend to exhibit slightly higher axial forces and normal stresses compared to asymmetric ones. Among the asymmetric configurations, the most favorable results are observed for structures labeled as structures B —where one corner is raised.

### 4.2. Type 2 Structures

Table 2 summarizes the results for type 2 structures, which feature internal bar arrangements forming planar meshes. As with type 1, all variants use identical cross-sectional profiles for both the load-bearing frame and internal grid, representing the minimum dimensions required for structural stability. The total mass of type 1 and type 2 structures is comparable, with differences not exceeding 4 kg. Within type 2, mass variation is minimal (up to 2 kg), making it an unreliable comparison metric. Maximum deflections are generally consistent, except in the reference model, which shows significantly higher deflection, bending moments, and shear forces. In other configurations, bending moments vary by no more than 0.05 kNm and shear forces by up to 0.1 kN. As in type 1, axial forces and normal stresses follow a discernible trend; however, in type 2, the trend is reversed: increasing the height difference between corners leads to a decrease in axial forces and normal stresses. Overall, these values are substantially higher than those observed in type 1 structures, indicating that mesh geometry has a pronounced influence on internal force distribution. Notably, mesh configurations with planar faces result in internal force values that are several times greater than those observed in configurations with non-planar faces.

### 4.3. Type 3 and 4 Structures

Table 3 and Table 4 present results for type 3 and 4 structures, characterized by reduced internal grids arranged in planes parallel to the vertical frame members, dividing them into four equal segments. Each type was analyzed under two geometric scenarios: corner lifting (A-1 to F-3) and corner lowering (G-1 to N-3), using identical minimum-profile bars for both frames and grids. Despite low internal forces, further profile reduction led to instability. Reference models (W-3 and W-4) consistently showed the highest deflections, bending moments, and shear forces. In both types, increasing corner height differences correlated with higher axial forces and normal stresses, while bending and shear remained relatively stable. When comparing types 3 and 4, changing the direction of internal bars resulted in a slight increase in internal forces, though the differences were marginal. Interestingly, when comparing types 3 and 4 with types 1 and 2, it was noted that despite the reduction in the number of structural elements, the overall structural mass increased—by more than 30 kg in some cases under identical loading conditions.

### 4.4. Summary and Comparative Evaluation of All Structural Models

To effectively visualize the results and support a comprehensive comparative analysis of the structural variants, selected outcomes are presented as line graphs in Figure 14, Figure 15, Figure 16, Figure 17, Figure 18, Figure 19, Figure 20, Figure 21, Figure 22 and Figure 23.

**Figure 14:**
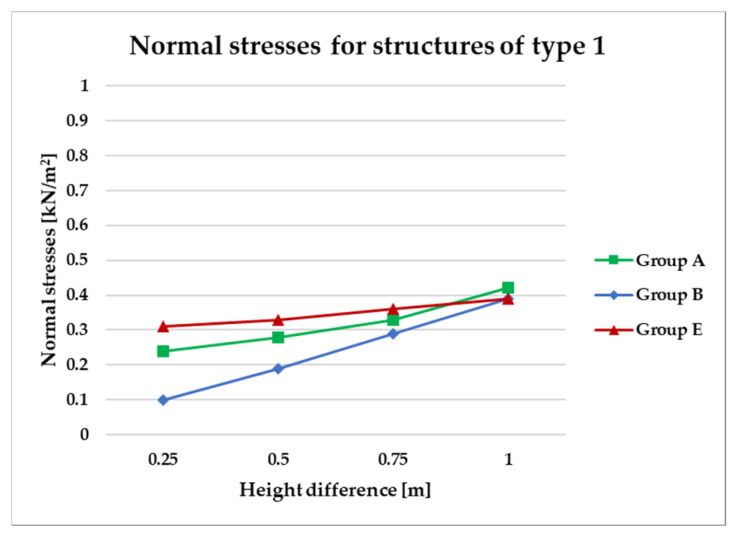
Normal stress diagram for structures of type 1.

**Figure 15:**
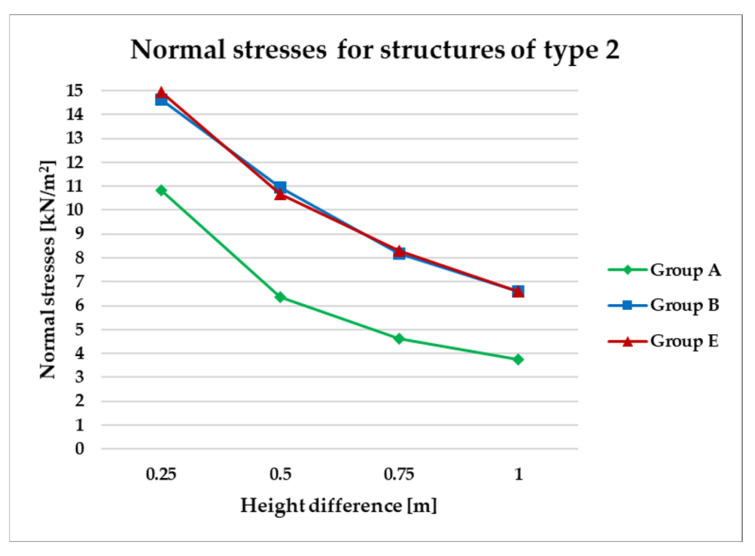
Normal stress diagram for structures of type 2.

**Figure 16:**
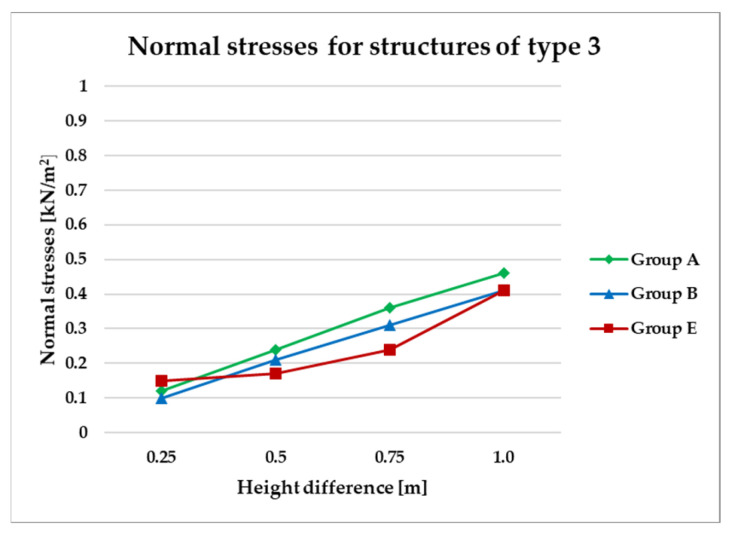
Normal stress diagram for structures of type 3.

**Figure 17:**
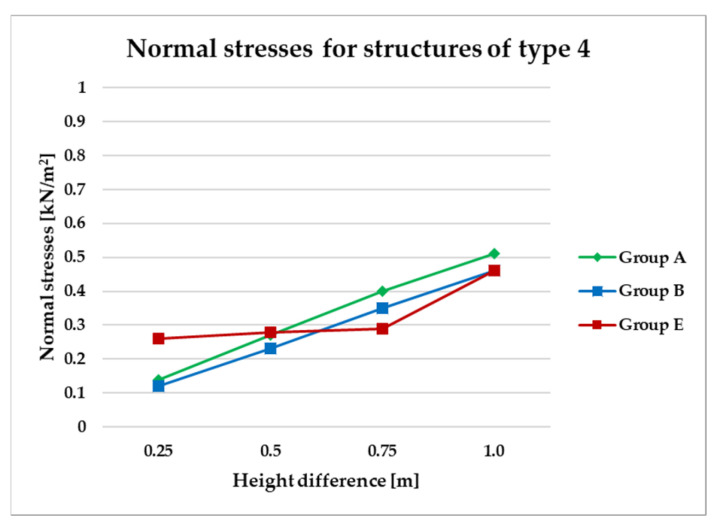
Normal stress diagram for structures of type 4.

Figure 14, Figure 15, Figure 16 and Figure 17 illustrate a comparison of normal stress values across structural groups within each structural topology. As is clearly shown in Figure 14, Figure 16 and Figure 17, structures of types 1, 3, and 4 exhibit a consistent increase in normal stress values with increasing height differentials between vertices. In contrast, Figure 15 reveals an inverse trend for type 2 structures, where greater height differences correspond to reduced normal stresses.

Figure 14 specifically depicts the distribution of normal stresses in type 1 structures, characterized by surfaces subdivided into non-planar quadrilateral fields. The analysis contrasts symmetrical configurations (Group A) with asymmetrical ones (Groups B and E). Among these, Group B demonstrated the most favorable structural performance, effectively minimizing normal stress concentrations. Conversely, Group E exhibited the least favorable results, with higher stress values indicating reduced structural efficiency.

However, Figure 15 presents the normal stress distribution for type 2 structures, comparing symmetrical configurations (group A) with asymmetrical ones (groups B and E). In this structural type—characterized by subdivision into planar quadrilateral fields—the most favorable stress performance was observed in the symmetrical group A. The remaining groups, B and E, exhibited comparable levels of structural efficiency, with no significant differences in stress behavior.

On the other hand, Figure 16 and Figure 17 present normal stress distributions for symmetrical (group A) and asymmetrical group B and E) configurations of type 3 and type 4 structures, respectively. In these structural types, the HP surface is subdivided by bar arrangements oriented in a single direction.

Both structural types type 3 and type 4 exhibit similar trends in stress behavior (Figure 16 and Figure 17).

**Figure 18:**
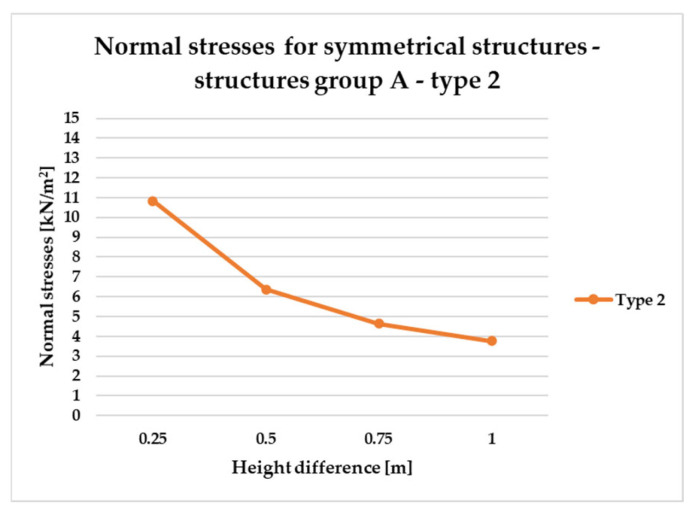
Normal stress diagram for symmetrical structures defined as A—raising of two frame corners—for all types.

**Figure 19:**
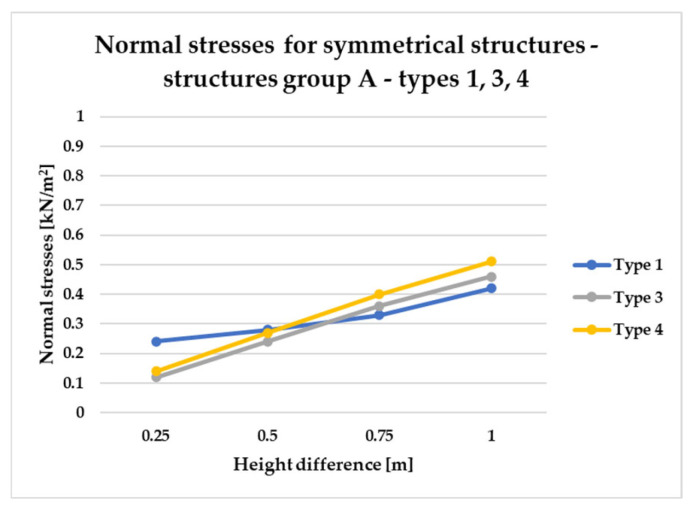
Normal stress diagram for symmetrical structures defined as A—raising of two frame corners—for types 1, 3, 4.

**Figure 20:**
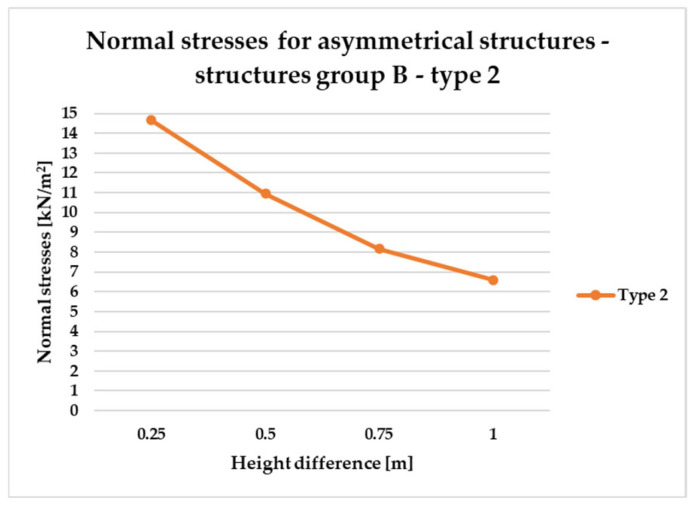
Normal stress diagram for asymmetrical structures (defined as B)—raising of one frame corner of type 2 structure.

**Figure 21:**
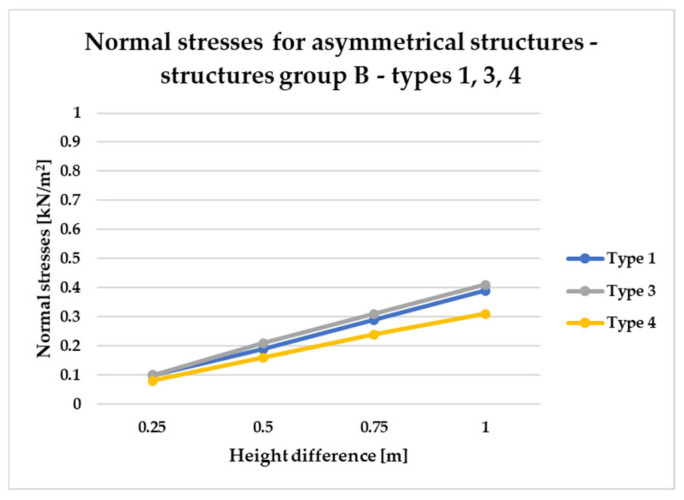
Normal stress diagram for asymmetrical structures (group B)—raising of a single frame corner—for types 1, 3, and 4.

**Figure 22:**
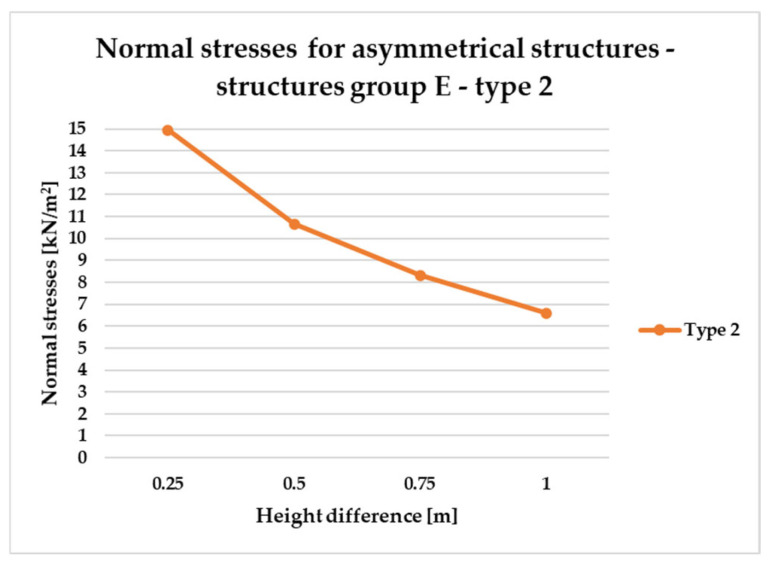
Normal stress diagram for asymmetrical structures (defined as E)—raising of two frame corners—for type 2.

**Figure 23:**
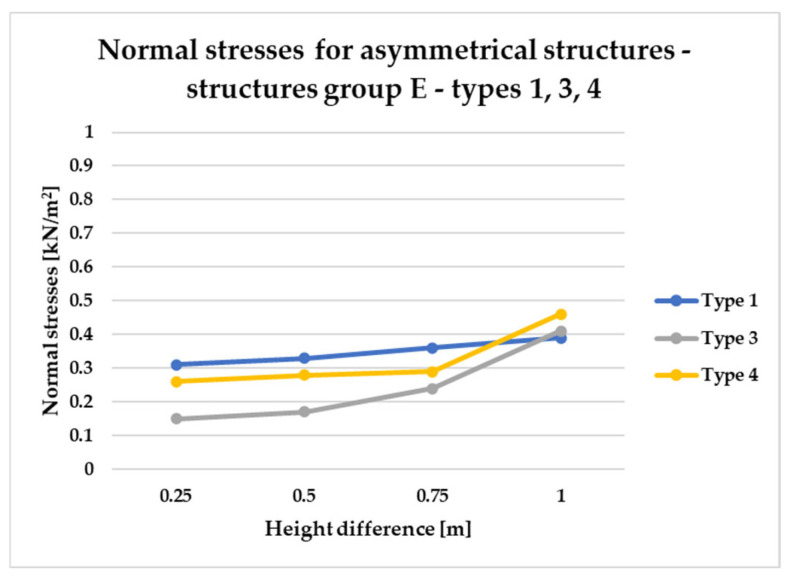
Normal stress diagram for asymmetrical structures (E)—raising of two adjacent frame corners—for types 1, 3, and 4.

To compare the results in terms of normal stresses for different types of structures, Figure 18, Figure 19, Figure 20, Figure 21, Figure 22 and Figure 23 present diagrams for structures with the same geometric shape but a different topology of the structural bar grid. The results for type 2 structures for all groups are presented in separate figures due to the significantly higher normal stress values compared to types 1, 3, and 4, which exhibited substantially lower stress levels.

The diagrams in Figure 18 and Figure 19 show behavior of symmetrical structures, defined as A, in which two opposite corners of the frame have been raised to the same height.

Figure 18 illustrates that as the height difference between the vertices of the type 2 structure (group A) increases, the corresponding stress magnitude decreases.

On the other hand, Figure 19 illustrates that the structural efficiency of group A configurations (types 1, 3, and 4) is influenced by variations in vertex elevation and the overall height of the structure. For geometries with minimal height differences, types 3 and 4 demonstrate superior efficiency, as indicated by lower normal stress levels. However, as the overall height increases, the efficiency of these types diminishes, while the performance of type 1 structures improves. Based on this analysis, it can be concluded that type 3 and 4 systems are more advantageous for low-rise structures, whereas type 1 configurations are better suited for taller forms within this group.

However, the results for asymmetrical structures belonging to group B, in which only one corner of the frame is elevated, are presented in Figure 20 and Figure 21.

In the case of asymmetric structures B of type 2, Figure 20, similarly to symmetrical structures (Figure 18), as the difference in height of the structure vertices increases, the magnitude of stresses decreases.

As shown in Figure 21, which presents internal force distributions for Group B across structural types 1, 3, and 4, type 4 structures consistently demonstrate the highest structural efficiency. This is evidenced by the lowest normal stress levels, regardless of variations in vertex height. While both type 1 and type 3 structures are characterized by similar stresses. Next, Figure 22 and Figure 23 present the results for asymmetric structural configurations, designated as Group E, in which two adjacent corners of the frame were rising.

Figure 23 illustrates that the structural efficiency of types 1, 3, and 4 within Group E is influenced by the magnitude of height differentials between adjacent vertices. For height variations up to 0.75 m, types 3 and 4 demonstrate superior performance, as indicated by lower normal stress values compared to type 1. However, at a height differential of 1.00 m, this trend reverses, with type 1 structures exhibiting reduced stress levels, thereby indicating higher structural efficiency within this range.

**Figure 24:**
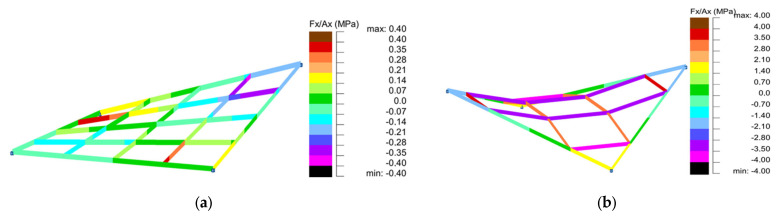
Maps of normal stresses for structures of (**a**) type 1 (B4); (**b**) type 2 (A4).

**Figure 25:**
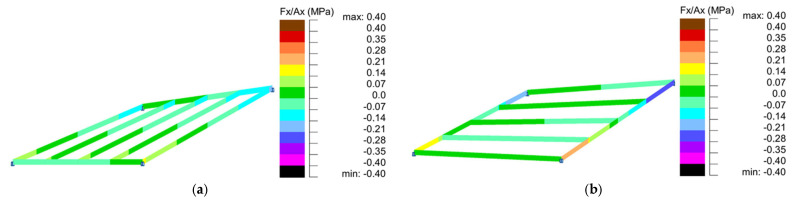
Maps of normal stresses for structures of (**a**) type 3 (E1); (**b**) type 4 (E1).

Based on the conducted analyses, the most pronounced differences in stress values were observed in type 2 structures, as illustrated in Figure 18, Figure 20 and Figure 22. These variations are notably greater when compared to the more consistent stress distributions found in types 1, 3, and 4, presented in Figure 19, Figure 21 and Figure 23. The analysis confirms that structural efficiency is strongly influenced by the topology of the internal bar system, which varies across structural types. Finally, Figure 24 and Figure 25 present the normal stress distribution maps for selected structurally efficient variants, highlighting the differences in stress behavior among the analyzed configurations.

At this stage, economic considerations could also be taken into account. Type 1 structures exhibit significantly lower mass compared to types 3 and 4, which may be a decisive advantage in practical applications.

### 4.5. Influence of Environmental Loads on the Behavior of Structures

The analyses conducted primarily focused on scenarios involving low, constant vertical loads. Nonetheless, cases involving climatic loads—such as snow and combined snow–wind conditions—were also examined and evaluated. Across these varied loading conditions, the structural response exhibited consistent behavior. Given the relatively low height of the analyzed structures, additional snow accumulation loads were not considered necessary. Furthermore, the geometric configuration of these structures does not support the formation of depressions that could facilitate significant snow buildup.

**Figure 26:**
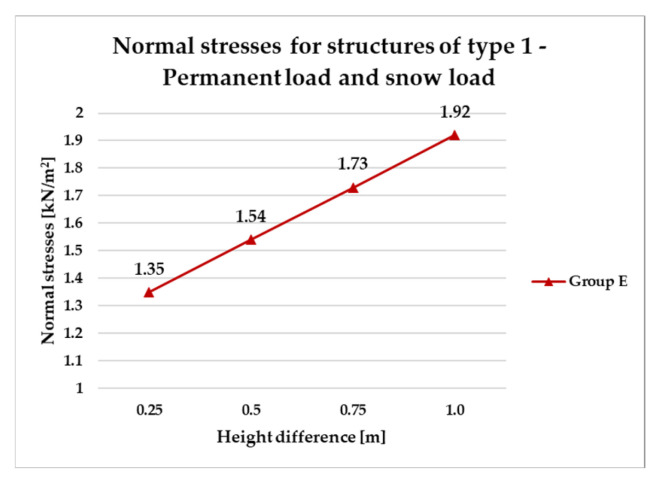
Normal stresses for structures of type 1—permanent load and snow load.

Figure 26 presents results for a sample group of structures of type 1 (E), designed taking into account the snow load equal to 1.20 kN/m^2^ for snow load zone III in Poland, which covers areas more exposed to heavy rainfall. For configurations subjected to evenly distributed permanent loads and vertical snow loads, the following cross-sectional profiles were adopted for the respective bar groups:

- Group G1: RO 63.5 × 4.0 mm;
- Group G2: RO 38.0 × 4.0 mm.

The utilization levels reached approximately 93% for the external frame members (G1) and around 73% for the internal structural grid bars (G2). The total structural mass increased to approximately 177 kg.

**Figure 27:**
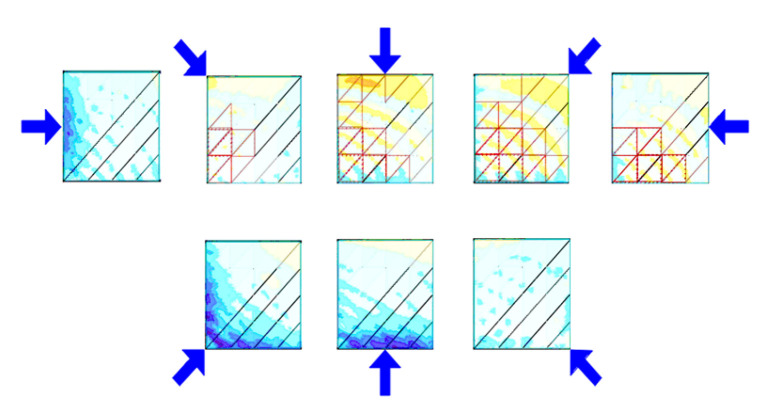
Considered wind directions and their impact on the roof.

To deepen the analysis and validate the trends observed within group E, the structural system was further evaluated under a combination of permanent loads, snow loads, and simulated wind loads. Climatic wind parameters were determined based on the conditions specific for zone I which covers the central part of Poland and is considered to have moderate wind load. Wind loads were generated automatically from eight directions with base velocity pressure equal to 0.25 kN/m^2^ (Figure 27).

**Figure 28:**
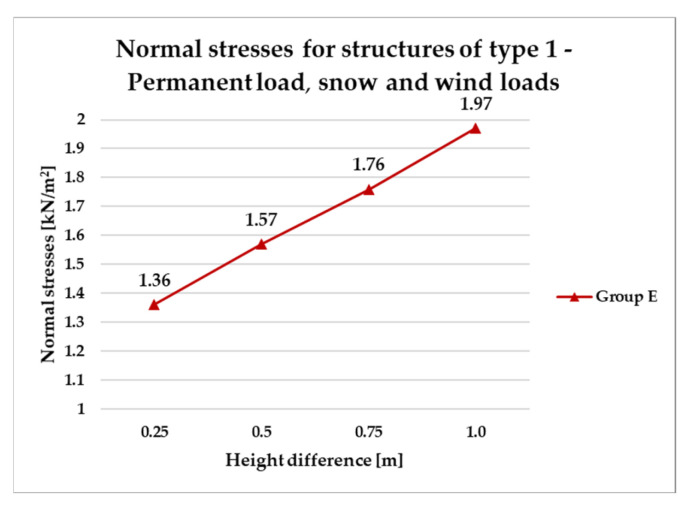
Normal stresses for structures of type 1—permanent load, snow and wind loads.

Based on the established assumptions, structural optimization was carried out with respect to mass efficiency. This process led to the selection of two cross-sectional profiles: G1—RO 70.0 × 4.0 mm, achieving an approximate bar utilization of 91%, and G2—RO 42.4 × 4.0 mm, with a utilization of approximately 71%. As a result of the optimization, the total structural mass increased to 198 kg. The corresponding outcomes, presented as normal stress values for the defined load cases, are illustrated in the line chart shown in Figure 28.

Following the generation of load combinations, it was determined that snow loading constitutes the dominant action for the analyzed geometries at the specified location. Consequently, the previously observed trend of increasing stress under prevailing vertical loads remains consistent. Therefore, the observed trends in the structure’s behavior presented in the previous sections should be maintained.

### 4.6. Practical Applications

The grid bar connections are primarily designed to be welded, while the outer grid bars may be bolted to the frame to facilitate assembly. The frame bar connections are also intended to be welded. However, for technological and practical reasons, both the grid and frame may incorporate assembly joints. Individual modules can be joined using connecting clamps, which are bolted to the outer frames.

In our study, strict boundary conditions were applied, including rigid joints and fixed supports at all four corners. Additionally, the structures were analyzed using semi-rigid connections between the frame and the bar grid. The introduction of these semi-rigid joints led to increased strain and higher utilization in the grid bars, while the utilization of the frame remained unaffected. Notably, these connections did not influence the cross-sectional areas of the bars.

**Figure 29:**
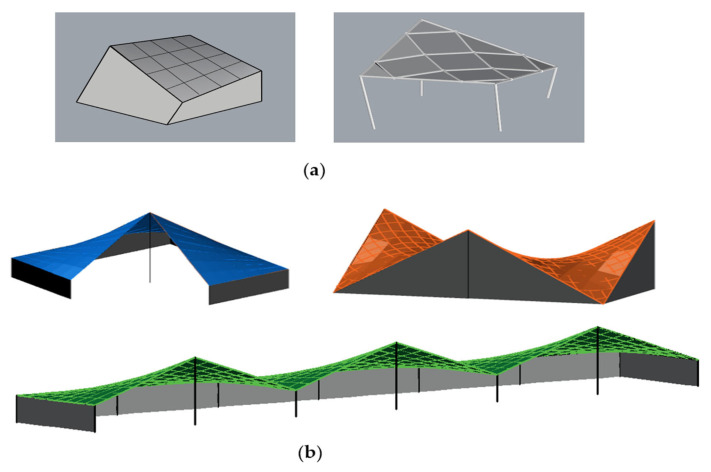
Examples of module roof structures based on HP: (**a**) single-module; (**b**) multi-module.

The findings presented in the previous sections can be directly applied to structures whose modules are supported at each corner, Figure 29a. In the case of more complex structural systems, the results remain applicable to configurations where the module arrangement does not promote snow accumulation.

Moreover, the obtained results can be helpful in shaping complex structures created by combining individual modules in both a linear and radial manner, like presented in [35,38]. The examples of such structures, both single-module and multi-module, are presented in Figure 29.

Further summary of the conducted research has been included in the Section 5.

## 5. Conclusions

This research presents a comparative analysis of the structural efficiency of steel bar modules based on HP geometry using Dynamo cooperating with ARSAP 2025 software for static and strength analysis, optimization, and dimensioning. The results obtained can be summarized in the following points.

- There is no universally optimal topology of the structural bar grid suitable for every HP geometry.
- The analysis results indicate that structural efficiency is strongly influenced by the geometry and topology of the internal bar system. For the modules with HP surface subdivisions into non-planar fields, asymmetric geometries were found to be the most effective, whereas for the modules with flat quadrilateral fields, symmetric configurations performed best.
- For low-rise configurations, a non-planar bar mesh proved to be more optimal, whereas for structures with significant vertex height variations, a planar mesh subdivision yields greater efficiency.
- Relatively low bending moments are observed at the nodes of the structural systems based on HP geometry, which positively influence the overall performance of the load-bearing systems.
- The obtained results showed that the observed trends in the behavior of the analyzed HP modules persists under climatic loads for the considered location.

The above findings, along with the observed design trends, provide valuable guidance for the design, optimization, and development of structural systems based on HP geometry.

It is important to note that the research conducted focused on a preliminary comparative analysis of module efficiency. For more complex structures—particularly those subject to snow accumulation or located in varying environmental conditions—a more detailed structural analysis is required. This should account for variable atmospheric loads, potential differences in support conditions, and connection methods. Nevertheless, the parametric definition of a single module enables the parametric modeling of an entire structure, allowing for rapid structural evaluation of the alternative design variants.

## Figures and Tables

**Table 1:** Results of the structural analysis for structures of type 1.

Structure Number	FU [%]	GU [%]	M [kg]	Max De [cm]	Max Di [cm]	Max My [kNm]	Max Fx [kN]	Max Fz [kN]	Max Fx/A [MPa]
W-1	16	36	138	0.7	2.0	0.43	0.00	0.57	0.00
A-1	16	36	139	0.4	0.3	0.45	0.06	0.59	0.24
A-2	16	36	140	0.4	0.3	0.45	0.08	0.57	0.28
A-3	16	34	140	0.4	0.3	0.44	0.13	0.56	0.33
A-4	16	34	142	0.4	0.3	0.44	0.17	0.55	0.42
B-1	16	36	139	0.4	0.3	0.44	0.04	0.57	0.10
B-2	16	36	139	0.4	0.3	0.44	0.07	0.57	0.19
B-3	16	37	140	0.4	0.3	0.45	0.11	0.57	0.29
B-4	16	35	141	0.4	0.3	0.45	0.15	0.58	0.39
C-1	16	35	141	0.4	0.3	0.45	0.15	0.57	0.41
C-2	16	36	141	0.4	0.3	0.49	0.16	0.57	0.51
C-3	16	34	141	0.4	0.3	0.44	0.17	0.56	0.43
D-1	16	35	139	0.4	0.3	0.44	0.08	0.57	0.21
D-2	16	35	140	0.4	0.3	0.44	0.12	0.57	0.31
D-3	16	35	140	0.4	0.3	0.44	0.12	0.56	0.33
E-1	17	37	140	0.4	0.3	0.45	0.12	0.58	0.31
E-2	17	37	140	0.4	0.3	0.45	0.13	0.58	0.33
E-3	17	35	140	0.4	0.3	0.45	0.14	0.58	0.36
F-1	16	36	139	0.4	0.3	0.44	0.07	0.57	0.17
F-2	16	36	140	0.4	0.3	0.45	0.09	0.57	0.24
F-3	16	36	140	0.4	0.3	0.45	0.10	0.57	0.27

**Table 2:** Results of structural analysis for structures of type 2.

Scheme	FU [%]	GU [%]	M [kg]	Max De [cm]	Max Di [cm]	Max My [kNm]	Max Fx [kN]	Max Fz [kN]	Max F/A [MPa]
W-2	15	34	136	0.5	2.0	0.42	0.00	0.55	0.00
A-1	20	26	136	0.2	0.3	0.20	4.05	0.3	10.83
A-2	14	17	137	0.1	0.1	0.17	2.39	0.26	6.36
A-3	11	14	138	0.1	0.1	0.16	1.72	0.24	4.63
A-4	10	12	139	0.1	0.1	0.15	1.40	0.23	3.76
B-1	28	44	136	0.2	1.0	0.27	5.43	0.38	14.65
B-2	21	27	136	0.2	0.3	0.20	4.11	0.30	10.95
B-3	17	20	137	0.2	0.1	0.18	3.11	0.27	8.16
B-4	15	18	138	0.1	0.1	0.17	2.50	0.26	6.59
C-1	13	15	138	0.1	0.1	0.16	2.08	0.25	5.44
C-2	12	14	138	0.1	0.1	0.16	1.77	0.25	4.68
C-3	11	13	138	0.1	0.1	0.15	1.56	0.24	4.16
D-1	16	20	136	0.2	0.1	0.18	3.04	0.27	8.03
D-2	14	17	137	0.1	0.1	0.17	2.44	0.26	6.43
D-3	13	15	137	0.1	0.1	0.16	2.02	0.25	5.36
E-1	30	47	138	0.3	1.0	0.28	5.68	0.39	14.95
E-2	18	28	137	0.1	0.4	0.13	4.04	0.18	10.67
E-3	17	20	137	0.2	0.1	0.18	3.19	0.27	8.31
F-1	28	45	136	0.2	1.0	0.28	5.50	0.28	14.74
F-2	29	46	137	0.2	1.0	0.28	5.58	0.39	14.84
F-3	21	27	137	0.2	0.3	0.20	4.19	0.30	11.04

**Table 3:** Results of structural analysis for structures of type 3.

Structure Number	FU [%]	GU [%]	Mass [kg]	Max De [cm]	Max Di [cm]	Max My kNm]	Max Fx [kN]	Max Fz [kN]	Max F/A [MPa]
W-3	26	11	155	1.4	1.1	1.23	0	1.58	0
A-1	26	11	156	0.8	0.6	0.71	0.09	0.90	0.12
A-2	26	11	156	0.8	0.6	0.7	0.17	0.90	0.24
A-3	25	11	157	0.8	0.6	0.7	0.25	0.88	0.36
A-4	25	11	159	0.7	0.6	0.69	0.33	0.87	0.46
B-1	25	11	156	0.8	0.6	0.71	0.07	0.91	0.1
B-2	25	11	156	0.8	0.6	0.71	0.14	0.91	0.21
B-3	25	11	157	0.8	0.6	0.71	0.22	0.91	0.31
B-4	25	11	158	0.8	0.6	0.71	0.29	0.92	0.41
C-1	25	11	157	0.8	0.6	0.71	0.3	0.91	0.42
C-2	25	11	158	0.8	0.6	0.70	0.31	0.90	0.44
C-3	25	11	158	0.7	0.6	0.69	0.32	0.88	0.45
D-1	25	11	156	0.8	0.6	0.71	0.16	0.91	0.23
D-2	25	11	157	0.8	0.6	0.71	0.23	0.91	0.33
D-3	25	11	157	0.8	0.6	0.70	0.24	0.90	0.34
E-1	20	8	158	0.6	0.6	0.55	0.10	0.72	0.15
E-2	20	8	158	0.6	0.6	0.55	0.12	0.72	0.17
E-3	20	8	158	0.4	0.6	0.54	0.17	0.72	0.24
F-1	20	8	156	0.5	0.6	0.54	0.06	0.71	0.08
F-2	20	8	157	0.6	0.6	0.54	0.08	0.71	0.11
F-3	20	8	157	0.5	0.6	0.54	0.11	0.71	0.16
G-1	20	8	156	0.5	0.5	0.54	0.07	0.70	0.09
G-2	20	7	156	0.5	0.5	0.54	0.13	0.70	0.18
G-3	18	9	157	0.5	0.5	0.53	0.19	0.69	0.27
G-4	20	8	159	0.5	0.5	0.53	0.25	0.68	0.35
H-1	20	8	156	0.5	0.5	0.54	0.05	0.71	0.08
H-2	20	8	156	0.5	0.5	0.54	0.11	0.71	0.16
H-3	20	8	157	0.5	0.5	0.54	0.17	0.71	0.24
H-4	20	8	158	0.5	0.5	0.54	0.21	0.71	0.31
K-1	20	8	157	0.5	0.5	0.54	0.23	0.71	0.32
K-2	20	8	158	0.6	0.5	0.59	0.26	0.78	0.37
K-3	20	8	158	0.5	0.5	0.53	0.24	0.69	0.34
L-1	20	8	156	0.5	0.5	0.54	0.12	0.70	0.17
L-2	20	8	157	0.5	0.5	0.54	0.18	0.70	0.24
L-3	20	8	157	0.5	0.5	0.54	0.18	0.71	0.26
M-1	20	8	158	0.6	0.5	0.55	0.10	0.72	0.15
M-2	20	8	158	0.6	0.5	0.55	0.12	0.72	0.17
M-3	20	8	158	0.5	0.5	0.54	0.17	0.72	0.24
N-1	20	8	157	0.6	0.5	0.54	0.08	0.71	0.11
N-2	20	8	157	0.6	0.5	0.54	0.08	0.71	0.11
N-3	20	8	157	0.5	0.5	0.54	0.11	0.71	0.16

**Table 4:** Results of structural analysis for structures of type 4.

Structure Number	FU [%]	GU [%]	Mass [kg]	Max De [cm]	Max Di [cm]	Max My [kNm]	Max Fx [kN]	Max Fz [kN]	Max F/A [MPa]
W-4	29	11	155	1.5	2.6	1.28	0	1.64	0
A-1	29	11	156	0.8	0.7	0.79	0.10	1.04	0.14
A-2	29	11	156	0.8	0.7	0.79	0.19	1.03	0.27
A-3	29	11	157	0.8	0.7	0.78	0.28	1.01	0.4
A-4	29	12	159	0.7	0.7	0.77	0.36	1.00	0.51
B-1	29	11	156	0.8	0.7	0.80	0.08	1.04	0.12
B-2	29	11	156	0.8	0.7	0.80	0.16	1.04	0.23
B-3	29	12	157	0.8	0.7	0.80	0.24	1.03	0.35
B-4	29	12	158	0.8	0.7	0.80	0.32	1.03	0.46
C-1	29	12	157	0.8	0.7	0.79	0.33	1.02	0.47
C-2	29	12	158	0.8	0.7	0.79	0.34	1.01	0.49
C-3	29	12	157	0.8	0.7	0.79	0.33	1.02	0.47
D-1	29	11	156	0.8	0.7	0.79	0.18	1.03	0.25
D-2	29	12	157	0.8	0.7	0.79	0.26	1.03	0.36
D-3	29	12	157	0.8	0.7	0.79	0.27	1.02	0.38
E-1	20	7	157	0.5	0.5	0.55	0.18	0.70	0.26
E-2	20	8	157	0.5	0.5	0.54	0.20	0.71	0.28
E-3	20	10	157	0.5	0.5	0.54	0.21	0.71	0.29
F-1	20	8	156	0.5	0.5	0.54	0.10	0.70	0.14
F-2	20	8	156	0.5	0.5	0.54	0.14	0.70	0.2
F-3	20	8	156	0.5	0.5	0.54	0.15	0.71	0.22
G-1	26	11	156	0.8	0.6	0.71	0.09	0.90	0.12
G-2	26	11	156	0.8	0.6	0.70	0.17	0.90	0.24
G-3	25	11	157	0.8	0.6	0.70	0.25	0.88	0.36
G-4	26	11	159	0.7	0.6	0.69	0.33	0.87	0.46
H-1	26	11	156	0.8	0.6	0.71	0.07	0.91	0.1
H-2	26	11	156	0.8	0.6	0.71	0.15	0.91	0.21
H-3	26	12	157	0.8	0.6	0.71	0.21	0.91	0.31
H-4	26	12	158	0.8	0.6	0.71	0.27	0.92	0.39
K-1	26	12	157	0.8	0.6	0.71	0.30	0.91	0.41
K-2	26	12	158	0.8	0.6	0.70	0.30	0.90	0.43
K-3	26	12	158	0.7	0.6	0.69	0.32	0.88	0.45
L-1	26	11	156	0.8	0.6	0.71	0.16	0.91	0.22
L-2	26	11	157	0.8	0.6	0.71	0.22	0.91	0.32
L-3	26	12	157	0.8	0.6	0.70	0.24	0.90	0.32
M-1	20	7	157	0.5	0.5	0.55	0.18	0.70	0.26
M-2	20	8	157	0.5	0.5	0.54	0.20	0.71	0.28
M-3	20	8	157	0.5	0.5	0.54	0.21	0.71	0.29
N-1	20	8	156	0.5	0.5	0.54	0.10	0.70	0.14
N-2	20	8	156	0.5	0.5	0.54	0.14	0.70	0.2
N-3	20	8	156	0.5	0.5	0.54	0.15	0.71	0.22

## Data Availability

The original contributions presented in this study are included in the article. Further inquiries can be directed to the corresponding author.

## Abbreviations

The following abbreviations are used in this manuscript:

ARSAP	Autodesk Robot Structural Analysis Professional
ULS	Ultimate Limit States
SLS	Serviceability Limit States
FU	Frame profiles utilization
GU	Grid profiles utilization
De	Bar deflections
Di	Node displacements
